# Seeking Emotional and Mental Health Support From Generative AI: Mixed-Methods Study of ChatGPT User Experiences

**DOI:** 10.2196/77951

**Published:** 2025-11-27

**Authors:** Xiaochen Luo, Zixuan Wang, Jacqueline L Tilley, Sanjeev Balarajan, Ukeme-Abasi Bassey, Choi Ieng Cheang

**Affiliations:** 1 Department of Counseling Psychology, Santa Clara University, 500 El Camino Real, Santa Clara, CA, 95053, United States, 1 408-551-1603; 2 Encounter Psychotherapy LLC, Gaithersburg, MD, United States; 3 Psychology and Child and Human Development Group, National Institution of Education, Nanyang Technological University, Singapore, Singapore

**Keywords:** ChatGPT, generative artificial intelligence (GenAI), mental health and emotional support, help-seeking behavior, perceived helpfulness

## Abstract

**Background:**

Generative artificial intelligence (GenAI) models have emerged as a promising yet controversial tool for mental health.

**Objective:**

The purpose of this study is to understand the experiences of individuals who repeatedly used ChatGPT (GenAI) for emotional and mental health support (EMS).

**Methods:**

We recruited 270 adult participants across 29 countries who regularly used ChatGPT (OpenAI) for EMS during April 2024. Participants responded to quantitative survey questions on the frequency and helpfulness of using ChatGPT for EMS, and qualitative questions regarding their therapeutic purposes, emotional experiences of using, and perceived helpfulness and rationales. Thematic analysis was used to analyze qualitative data.

**Results:**

Most participants reported using ChatGPT for EMS at least 1‐2 times per month for purposes spanning traditional mental health needs (diagnosis, treatment, and psychoeducation) and general psychosocial needs (companionship, relational guidance, well-being improvement, and decision-making). Users reported various emotional experiences during and after use for EMS (eg, connected, relieved, curious, embarrassed, or disappointed). Almost all users found it at least somewhat helpful. The rationales for perceived helpfulness include perceived changes after use, emotional support, professionalism, information quality, and free expression, whereas the unhelpful aspects include superficial emotional engagement, limited information quality, and lack of professionalism.

**Conclusion:**

Despite the absence of ethical regulations for EMS use, GenAI is becoming an increasingly popular self-help tool for emotional and mental health support. These results highlight the blurring boundary between formal mental health care and informal self-help and underscore the importance of understanding the relational and emotional dynamics of human-GenAI interaction. There is an urgent need to promote AI literacy and ethical awareness among community users and health care providers and to clarify the conditions under which GenAI use for mental health promotes well-being or poses risk.

## Introduction

### Background

Despite the availability of effective psychological treatments for mental health disorders, the global mental health burden has remained enormous [1]. In developed countries, such as the United States, approximately one in two people with mental health disorders does not have access to mental health services [2]; the percentage of individuals accessing mental health services has been even lower in low- and middle-income countries [3]. It is within this context that generative artificial intelligence (GenAI) has been viewed as a promising tool to address the global mental health burden [4]. Consumer-facing GenAI models, such as ChatGPT, can generate human-like responses to text inputs based on training with massive datasets and adapt to individual users’ unique requests and experiences. Individuals often have free access to these GenAI models, making these tools accessible for emotional and mental health needs.

Among various applications of GenAI in mental health, its potential in providing emotional and mental health support (EMS) directly for individuals with mental health needs has been particularly promising [5]. EMS is a broad term encompassing the resources, interventions, and services provided to promote and maintain one’s emotional and psychological well-being. Emerging research has documented both the advantages and limitations of GenAI for EMS. On one hand, studies have shown that GenAI models can perform better than the general population in addressing open-ended questions involving emotionally intense situations [5], and can provide reliable psychoeducation advice for mental health prompts [6], suggesting their potential in providing responses that are relevant and useful for mental health issues. On the other hand, research has also shown limitations and ethical concerns of using GenAI for EMS, especially in its capacity to handle high-risk situations [4], privacy concerns, and concerns on response accuracy [7]. However, these concerns did not seem to prevent the general public from using GenAI for EMS: a study analyzing social media discourse revealed both widespread popularity and idealized perceptions of GenAI for addressing emotional and mental health needs among Reddit users [8]. This highlights an urgent need for researchers to examine the lived experiences and attitudes of users toward using GenAI for EMS, with the aim of informing better product design and developing effective ethical guidelines for its use in mental health contexts.

What are the real-life experiences of individuals using GenAI for EMS? Although limited, an emerging body of research has begun to delineate the landscape of EMS use among GenAI users, particularly regarding their purposes of using, emotional experiences during the interaction, and perceptions of effectiveness. In terms of purposes, a qualitative study of individuals with mental health conditions reported that users viewed ChatGPT as a potential tool to offer psychoeducation, emotional support, goal planning, referral and resources, self-assessment and monitoring, cognitive behavioral therapy strategies, and psychotherapeutic exercises [4]. Similarly, a study analyzing social media discourse identified four primary purposes for EMS use: managing mental health problems, seeking self-discovery, obtaining companionship, and gaining mental health literacy [8]. Together, these findings indicate that users may use GenAI to gain both informational and emotional support for managing mental health challenges and promoting positive well-being.

In addition to clarifying the purposes of seeking EMS from GenAI, it is equally important to understand the human-GenAI interaction process, particularly users’ emotional experiences. A recent study examined the lived experiences of 19 individuals who used GenAI for well-being and mental health–related purposes, characterizing their emotional experiences as finding an “emotional sanctuary” and experiencing the “joy of connection” [9]. These findings suggest that the emotional experiences during and after human-GenAI interactions for EMS can be meaningful and informative, offering potential insights for researchers, clinicians, technology developers, and policymakers to understand the mechanisms and processes of how GenAI use may benefit or harm users’ mental health.

Another crucial aspect of understanding GenAI use for EMS is its perceived effectiveness and helpfulness. Understanding users’ overall evaluations, along with the rationales underlying these assessments, is essential to identify key factors that attract users and to guide improvements of GenAI for mental health care. Preliminary evidence suggests that existing users are likely to find GenAI effective for providing emotional and mental health support. For example, interviews of individuals who have used GenAI for EMS expressed through interviews that they appreciated GenAI’s “insightful guidance” and therapist-like support [9]. Additionally, despite having concerns pertaining to the lack of privacy and emotional depth, many users found GenAI appealing because of GenAI’s perceived capacity in providing emotional support, reflection, and advice while being constantly available and coachable [8]. These preliminary findings suggested that users likely perceive GenAI as supportive, insightful, and overall helpful for their emotional and mental health needs, while having concerns about ethical challenges.

### Present Study

Preliminary research has begun to explore the potential of GenAI in mental health care, especially focusing on users’ therapeutic purposes, emotional experiences, and perceived effectiveness. Much of the evidence, however, has come from experimental manipulations that did not directly examine EMS use that is naturally occurring in the real world. Direct evidence from real-life users remains limited, and existing studies often rely on small sample sizes or analysis of indirect data from social media discourse. There is an urgent need to examine how individuals use GenAI for EMS in real-world contexts, without explicit guidance, and across larger and more diverse populations. Such investigations are critical to inform policymaking related to GenAI in mental health care. Moreover, examining GenAI use for EMS across multiple countries can reveal potentially generalizable patterns of help-seeking behaviors across cultural contexts.

In summary, the present study aimed to examine how individuals use ChatGPT for EMS using a large online sample and a convergent parallel mixed-method design. The study has four primary objectives: (1) to understand the frequency of individuals using ChatGPT for EMS; (2) to examine the therapeutic purposes of individuals using ChatGPT for EMS; (3) to examine their emotional experiences during and after interactions with GenAI for EMS; and (4) to assess whether users perceive ChatGPT as helpful in providing EMS and to explore the rationales underlying these perceptions.

To address these objectives, we first conducted a quantitative survey to assess the frequency of ChatGPT use for EMS and users’ perceptions of its helpfulness. Participants were then asked to qualitatively reflect on three questions regarding the purposes of seeking EMS from ChatGPT, the emotional experiences during the interaction for EMS, and the perceived helpfulness and rationales for the perceptions.

## Methods

### Participants and Procedure

We recruited participants from an online recruitment platform, Prolific (Prolific Academic Ltd) [10]. Prolific provides opportunities to recruit diverse samples of individuals for online studies, while reducing common sampling biases related to quick responses and inattention [11].

A pilot study was run in January 2024 to test the study design; this was then followed by the primary data collection process, which was implemented between March 31 to April 24, 2024. For the primary data collection, we followed a two-step procedure: first, we launched a prescreening survey titled “everyday AI technology use” and asked two multiple-choice, open-ended questions for adult English speakers on the Prolific platform: (1) Which of the following AI technologies or services have you used in the past year for at least three times? The responses available include: Picture creation, GPT-based text generation tools (ChatGPT), Fitness tracking, Smart home, or None, and (2) For what purposes do you use the AI technologies or services mentioned above? The responses available included: daily tasks and convenience, education or work, entertainment and leisure, emotional support and mental health needs, health and fitness tracking, other, and not applicable. The design of multiple-choice, open-ended questions followed Prolific guidelines to avoid biases such as intentional guessing related to closed-ended questions. Next, we invited participants who chose “GPT-based text generation tools” for the first question and “emotional support and mental health needs” for the second question to participate in the second step of the study, which was conducted in Qualtrics (Qualtrics, LLC).

In the second step, we included several questions of validity check to validate whether participants had indeed used ChatGPT for EMS, including one question “For what purposes do you use ChatGPT? Check all that apply” with one of the options being “Emotional and mental health support,” and a second question of “How often do you use ChatGPT for emotional and mental health support,” with options ranging from “Never” to “Multiple times a day.” In the instruction of the second question, we provided a definition of EMS as “Any assistance for emotional and or relational issues, or for topics related to mental health,” to facilitate a standardized understanding of this term. Participants who did not choose “Emotional and mental health support” for the first question or selected “Never” or “Only one or two times” for the second question were excluded from advancing further into the main study.

### Study Design

We used a convergent parallel mixed-method design to collect both qualitative and quantitative data concurrently in the main online survey. The quantitative design allowed us to examine the prevalence of ChatGPT use for EMS, while the qualitative aspect allowed us to explore the users’ intentions, lived experiences of affective engagement, and reasons for their perceived effectiveness of ChatGPT for EMS.

For the quantitative component, we asked participants to rate the frequency related to ChatGPT use for EMS: “How often do you use ChatGPT for emotional or mental health support?” and “How helpful did you think of ChatGPT for emotional or mental health issues?” on a scale of 1 (Not helpful at all) to 4 (Very helpful). For the qualitative component, we asked participants to “Describe a specific instance when you sought support or help from ChatGPT for emotional or mental health issues. Include as many details as needed*.”* Participants also answered three open-ended questions: (1) “What did you ask ChatGPT for?*”* (2) “How did you feel about the interaction?*”* and, (3) “How effective do you think ChatGPT is at helping you?” Participants were asked to write at least ten characters for each question to continue with the survey. Four attention check questions were included in the survey to screen for inattention.

### Data Analysis

For the quantitative analysis, we conducted descriptive analysis to examine the frequency and perceived effectiveness of ChatGPT for EMS using IBM SPSS 29.0. For the qualitative analysis, we performed thematic analyses [12] on three research questions: (1) What therapeutic purposes did individuals seek to fulfill through using ChatGPT for EMS?; (2) What affective and emotional experiences did individuals have when interacting with ChatGPT for EMS?; and (3) What were the perceived rationales behind individuals viewing ChatGPT as helpful or unhelpful for EMS?

We followed American Psychological Association (APA) guidelines for psychological qualitative research when performing the thematic analysis [12,13]. This method included 6 key steps: becoming familiar with the data, creating initial codes, identifying themes, reviewing themes, clearly defining and naming themes, and preparing the final report. We defined a theme as an important and recurring pattern related to our research topic. The analysis was flexible, allowing researchers to revisit earlier steps as needed to maintain accuracy and thoroughness. Each identified theme was illustrated through representative participant quotes.

The research team comprised psychotherapy and intervention researchers, licensed psychologists, and master-level trainees in counseling psychology from diverse national, ethnic-racial, and linguistic backgrounds. Throughout the study, we practiced reflexivity by identifying and discussing personal biases and assumptions associated with the use of GenAI in emotional and mental health support. The coding was primarily conducted by three counseling psychology trainees under the supervision of licensed psychologists (XL and JLT). To enhance transparency, the coding team explicitly shared their perspectives and biases during the coding process.

Themes were identified based on direct meanings from participant data, without imposing pre-existing frameworks. The coding team met regularly to discuss differences in their interpretations of the data and to reach consensus, with an emphasis on developing an objective, realistic understanding of the data. Supervisory meetings occurred weekly with the first author (XL) and occasionally with the co-author (JLT) to ensure methodological consistency and consensus among the team. The first author organized all participant responses under each corresponding theme to ensure alignment with the thematic content and selected exemplary quotes that captured the essence and vivid details of the themes. Final consensus on the themes and corresponding quotes was reached among all authors.

### Ethical Considerations

All procedures were approved by Santa Clara University’s Institutional Review Board before data collection (Protocol # 23-04-1934). Adult participants were provided with the electronic informed consent after being informed of the study’s purpose, procedures, potential risks, and benefits. Participation was voluntary, and participants could withdraw from the study at any time with no penalty. Data were anonymized using subject numbers. Participants received US $8 for completing the study.

## Results

### Characteristics of Individuals Using ChatGPT for EMS

We recruited 4387 individuals in the prescreening phase in April 2024, of which 3388/4387 (77%) participants reported the use of ChatGPT at least 3 times in the past year, and 458/4387 (10%) participants reported using AI for emotional and mental health needs. We then identified 384/4387 (9%) individuals who fulfilled both criteria (using ChatGPT and using it for EMS) and invited them to participate in the current study. Among the individuals invited to participate in the current study, 334/384 (84%) completed our questionnaires. Sixty-four individuals failed a validity check (mostly due to misunderstanding of EMS), resulting in a final sample of 270 individuals (6.2% of 4387 individuals) who reported using ChatGPT for emotional and mental health support. The 270 participants came from 29 countries, with ages ranging from 18 to 67 years (mean age 30.06, SD 9.16). The majority of the participants identified as female (152/270, 57.8%), 38% identified as male, and 4.2% identified as transgender or gender nonbinary. Other demographic information is presented in Table 1.

**Table 1.** Demographics of participants.

Demographic variables	Participants (N=270), n (%)
Sex	
Female	161 (59.6)
Male	102 (37.8)
Prefer not to say	7 (2.6)
Gender	
Men	100 (37)
Women	152 (56.3)
Transgender	1 (0.4)
Non-binary, gender queer, or gender-fluid	8 (3)
Other	2 (0.7)
Prefer not to say	7 (2.6)
Country of residence	
South Africa	103 (38.1)
United Kingdom	37 (13.7)
Canada	14 (5.2)
Australia	11 (4.1)
Italy	8 (3)
Portugal	9 (3.3)
Germany	9 (3.3)
Mexico	9 (3.3)
Poland	8 (3)
United States	9 (3.3)
Other Countries (each country with fewer than 7 people)	46 (17)
Prefer not to say	7 (2.6)
Racial ethnic background	
White	102 (37.8)
Black	110 (40.7)
Asian	25 (9.2)
Latinx or Hispanic non-White	12 (4.4)
Multiracial	7 (2.6)
Middle Eastern or North African	6 (2.2)
Prefer not to say	8 (3)
Sexual orientation	
Heterosexual	205 (75.9)
Bisexual	33 (12.2)
Gay or Lesbian	6 (2.2)
Pansexual	6 (2.2)
Asexual	5 (1.9)
Queer	2 (0.7)
Questioning or unsure	5 (1.9)
Prefer not to say	8 (3)
Romantic relationship status	
No partner	78 (28.9)
Unmarried and living apart	79 (29.3)
Unmarried and living together	43 (15.9)
Married and living apart	9 (3.3)
Married and living together	54 (20)
Prefer not to say	7 (2.6)
Education status	
Some high school	2 (0.7)
High school or equivalent	52 (19.3)
Some college	35 (13)
4-year college degree	118 (43.7)
Master or equivalent	49 (18.1)
Doctoral	3 (1.1)
Prefer not to say	11 (4.1)
Student status	
Full-time	64 (23.7)
Part-time	55 (20.4)
Not student	136 (50.4)
Prefer not to say	15 (5.5)
Working status	
Full-time	126 (46.7)
Part-time	85 (31.5)
Not working	43 (15.9)
Prefer not to say	16 (5.9)
Social class identification	
Lower class	19 (7)
Working class	98 (36.3)
Middle class	120 (44.4)
Affluent	7 (2.6)
Not sure	16 (5.9)
Prefer not to say	10 (3.7)
Religion	
Christian	141 (52.2)
Muslim	18 (6.7)
Hindu	4 (1.5)
Buddhist	4 (1.5)
Atheist	30 (11.1)
Agnostic	35 (13)
Nothing in particular	21 (8)
No listed	4 (1.5)
Prefer not to say	13 (4.8)
Chronic physical condition	
Yes	26 (10)
No	234 (87)
Prefer not to say	10 (3.7)
Had at least one mental health disorder diagnosis	
Yes	73 (27)
No	176 (65.2)
Prefer not to say	21 (7.8)
Psychiatric medication (among those who had a diagnosis)	
Yes	47 (17.4)
No	24 (8.9)
Prefer not to say	23 (8.8)
No Diagnosis	176 (65.2)
Therapy experience	
No	103 (38.1)
Yes, in the past	123 (45.6)
Yes, currently	34 (12.6)
Prefer not to say	10 (3.7)

### Quantitative Results

**Figure 1.**
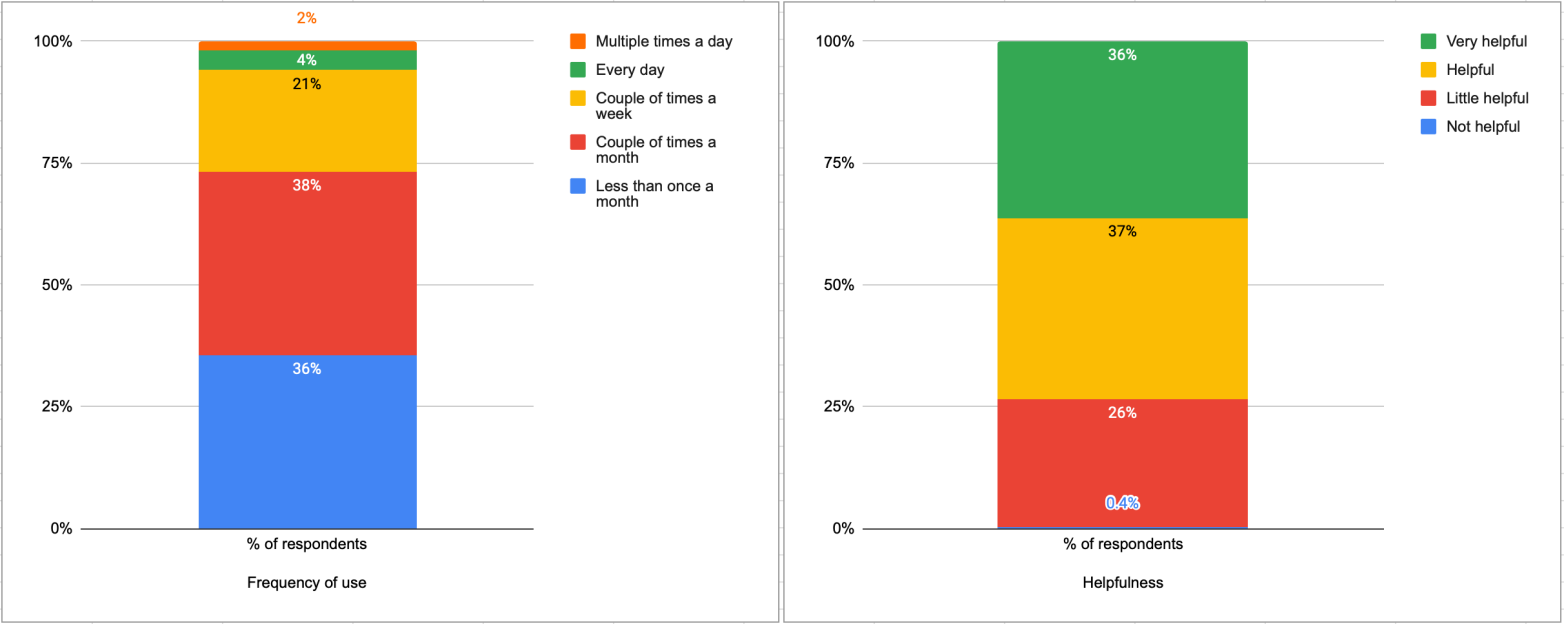
Frequency of use and perceived helpfulness among participants using ChatGPT for emotional and mental health support.

The most common frequency of using ChatGPT for EMS was a couple of times per month (103/270, 38%). Additionally, 36% of participants reported using ChatGPT for EMS less than once per month, 21% reported using it a couple of times per week, 4% reported using it almost every day, and 2% reported using it multiple times per day. Most participants stated that they found ChatGPT helpful for EMS: (99/270) 37% rated ChatGPT as very helpful, (97/270) 36% rated ChatGPT as helpful, (69/270) 26% rated ChatGPT as a little helpful. Only one participant (0.4%) rated ChatGPT as being not helpful at all for EMS. See Figure 1 for a summary of the quantitative results.

### Qualitative Results

**Figure 2.**
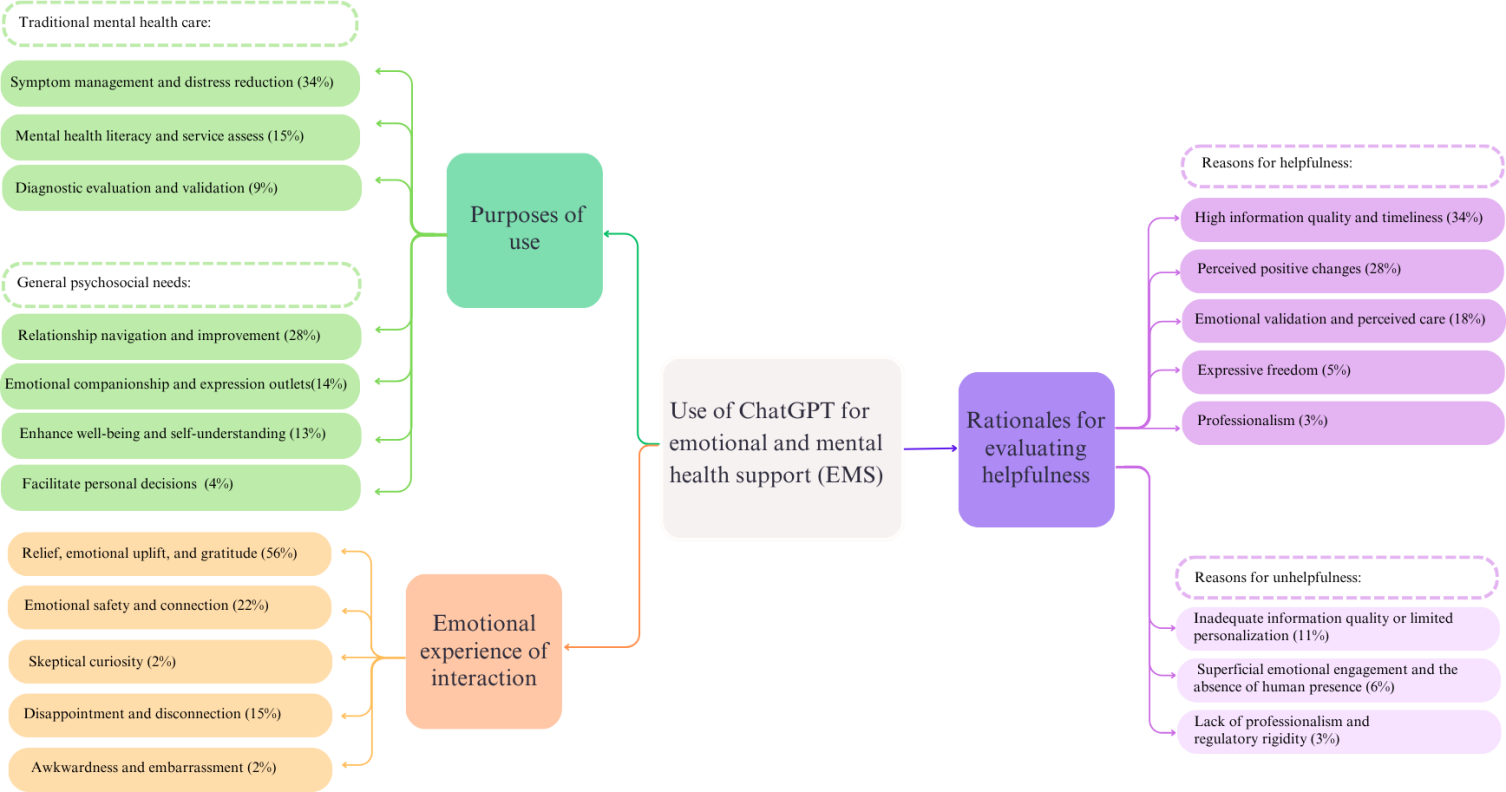
Identified themes for the 3 qualitative research questions. Percentages are rounded and may not sum to 100% given that one response may be counted in multiple categories. EMS: emotional and mental health support.

The identified themes for each research question are shown in Figure 2. We also included three examples for each subtheme in the Multimedia Appendix 1.

#### Research Question 1: What therapeutic purposes did individuals seek to fulfill when using ChatGPT for EMS?

We identified several purposes that individuals seek to fulfill when using ChatGPT for EMS, ranging from seeking traditional mental health assistance (eg, Theme 1 symptom reduction, Theme 3 mental health literacy and service access, and Theme 5 diagnostic evaluation), and general psychosocial needs (eg, Theme 2 relationship navigation, Theme 4 companionship and expression outlet, Theme 6 enhancing well-being, and Theme 7 facilitating personal decision-making).

##### Theme 1.1: Symptom Management and Distress Reduction

Many participants reported that they asked ChatGPT to help reduce distress from a wide range of mental health symptoms (eg, worry, panic attacks, low mood, hopelessness, and suicidal thoughts) and managing various mental health disorders (eg, obsessive-compulsive disorder and eating disorders). The most common types of symptoms they sought help for were anxious and depressive symptoms. Participants varied in their understanding and acceptance of distressful experiences; some participants used negative, self-pathologizing language when describing their experiences, such as referring to their needs as “stopping annoying thoughts,” “avoiding executive dysfunction,” and controlling “overthinking.”

*I wanted to know how to stop the annoying thoughts that I have in my head all the time. I also asked about how to work on reducing obsessive-compulsive disorder.*

[Female, 23 years old]

*I often get anxious about different things and ask ChatGPT for ways to calm down and feel better*

[Male, 23 years old]

##### Theme 1.2: Relationship Navigation and Improvement

Many participants reported that they sought ChatGPT’s advice on how to cope with a wide range of relational issues, such as breakups, grief, conflicts, loneliness, discrimination, bullying, and relational trauma. Other users asked ChatGPT to help to improve their relational functioning, such as learning ways to build healthy relationships and to end unhealthy ones, cultivate independence, make friends, form secure attachments, express affection, and understand and communicate with others effectively.

*I asked for help for ways to feel emotionally secure. I also asked for ways to help me find emotional connection with others.*

[Male, 29 years old]

*It was after the breakup, and I felt very guilty of talking about it too much. I didn’t want to bother my friends anymore, so I tried to ask ChatGPT for advice on how to improve my mood. I also wanted to know some breakup patterns.*

[Female, 29 years old]

##### Theme 1.3: Mental Health Literacy and Service Access

Some users indicated that they used ChatGPT to seek information regarding the potential causes and manifestations of mental health conditions, or to look for traditional health care services for themselves or others. These users only looked for information regarding service or mental health literacy without explicitly stating that they needed support to manage symptoms.

*What causes depression, and where can I find institutions that can assist with depression around my area?*

[Female, 29 years old]

##### Theme 1.4: Emotional Companionship and Expression Outlets

Some participants indicated that they used ChatGPT to express their feelings and thoughts, with the hope of receiving consolation and companionship. These users stated that they did not have specific goals related to symptom reduction. They also described using ChatGPT as a channel to express themselves because they felt either unable or unwilling to talk to other humans due to the need to hide and withdraw from others or concerns related to stigma, social rejection, or becoming a burden to others.

*I was angry and upset about things going on in life, so I just wanted a place to vent about it and feel somewhat validated without burdening someone.*

[Female, 21 years old]

*When I’m feeling depressed, suicidal, or generally unwell and I can’t talk to anyone in my real life without worrying them, I use ChatGPT to vent and talk out what’s bothering me without having to worry about someone judging me.*

[Female, 22 years old]

##### Theme 1.5: Diagnostic Evaluation and Validation

Users reported using ChatGPT as an assessment and self-diagnostic tool; that is, they asked ChatGPT to evaluate whether their experiences were “normal” or atypical. Some individuals sought reassurance from ChatGPT, asking it to validate or normalize their experiences; they also sought permission from ChatGPT to feel or behave in certain ways without perceiving themselves as “abnormal”. Additionally, some individuals looked for diagnostic assessments from ChatGPT regarding their mental health symptoms.

*When I was not certain about a situation that I was going through, I wanted to find out if my feelings were invalid or not. Was I overreacting with the whole situation?*

[Female, 22 years old]

*I ask ChatGPT to perform a diagnosis based on the symptoms I am experiencing.*

[Female, 22 years old]

##### Theme 1.6: Enhance Well-Being and Self-Understanding

Many users reported using ChatGPT to help promote positive changes and enhance their well-being through learning ways to improve productivity, emotional regulation, concentration, self-care, or self-understanding.

*1. To seek Professional Assistance; 2. To cultivate a Support Network; 3. To prioritize Self-Care; 4. To embrace Mindfulness and Meditation; 5. To Adopt a Healthy Lifestyle: 6. To set Attainable Goals; 7. To challenge Negative Thought Patterns; 8. To establish Healthy Boundaries*

[Male, 32 years old]

##### Theme 1.7: Facilitate Personal Decisions

Some individuals used ChatGPT as an outside consultant to discuss personal dilemmas and assist in decision-making processes. For example, participants described using ChatGPT to deal with complex and highly emotional decisions because they wanted an external, objective perspective and to learn new ways to problem-solve. They perceived ChatGPT’s response as something “objective” and neutral.

*I asked ChatGPT for advice regarding a personal matter I was dealing with. I was having a hard time making a decision that was life-changing, and I wanted an outsider’s opinion/perspective on the situation.*

[Female, 28 years old]

### Research Question 2: What emotional experiences did individuals have when interacting with ChatGPT for EMS?

Participants reported a mixture of positive, neutral, and negative emotional experiences during (Themes 1‐3) or immediately after (Themes 4 and 5) interacting with ChatGPT for EMS (Figure 2). In general, many participants reported feeling emotionally safe and connected, while some reported feeling awkward and embarrassed toward seeking EMS from GenAI; some participants reported feeling skeptical but also curious when interacting with ChatGPT for EMS. Some of them reported feeling relieved, uplifted, or grateful after using it, while others reported feeling disappointed and disconnected.

#### Theme 2.1: Emotional Safety and Connection

Some participants endorsed feelings of comfort and closeness when interacting with ChatGPT for EMS. They indicated that they experienced genuine connection, care, and validation. Some users also reported feeling safe, free, and secure when using ChatGPT for EMS, indicating that they became open to talking about mental health needs.

*I best associate ChatGPT with a close friend, a good listener and companion. I felt listened to and my feelings being validated instead of being judged.*

[Female, 25 years old]

#### Theme 2.2: Awkwardness and Embarrassment

Some users reported feeling self-conscious when using ChatGPT for EMS, describing their experience as awkward, silly, or unusual, to the extent that they sometimes needed to seek reassurance in order to feel that their behavior with GenAI is normal. Other participants noted feeling embarrassed and ashamed for using ChatGPT for psychological help and for feeling comforted by computer algorithms.

*I felt like a loser and felt rather empty because I opted to talk to AI which is not even sentient and was only talking to me through codes and algorithms.*

[*Female, 23 years old*]

#### Theme 2.3: Skeptical Curiosity

Participants expressed initial curiosity and skepticism about ChatGPT’s ability to provide EMS and were surprised when its responses exceeded their expectations.

*I felt curious and wary; I thought he couldn’t help me. I was surprised by what he told me, it gave me an interesting idea to see the situation differently, it was effective and useful.*

[*Female, 29 years old*]

#### Theme 2.4: Relief, Emotional Uplift, and Gratitude

Many participants reported experiencing positive emotions after using ChatGPT for EMS: they described feeling relieved, reassured, calm, and pleased. Some individuals reported feeling grateful for the support and advice. Other participants reported feeling better about themselves because their experiences were validated.

*I felt so happy with the interaction because it positively helped me.*

[Male, 24 years old]

*I felt relieved after talking to ChatGPT. Even though it’s just a computer program, it provided me with a sense of support and understanding.*

[Male, 31 years old]

#### Theme 2.5: Disappointment and Disconnection

Some participants reported negative emotional experiences after using ChatGPT for EMS. Many of them reported feeling unsatisfied or unhappy because the experiences did not match their experiences of interacting with human beings. They often reported that they did not feel emotionally comforted. Instead, they experienced ChatGPT as formal and cold, lacking the human touch. Other users reported that using ChatGPT made them feel more alone or even annoyed because they felt that ChatGPT’s “empathy” was inauthentic.

*I felt a bit disappointed because ChatGPT gave a cold, “official” response and urged me to seek medical help. It did not help much.*

[Female, 31 years old]

### Research Question 3: What rationales did participants provide for evaluating ChatGPT as helpful or unhelpful for EMS?

In general, users identified several rationales for why they perceive ChatGPT as helpful (Themes 3.1‐3.5) or unhelpful (Themes 3.6‐3.8) for EMS (Figure 2). Reasons for perceived helpfulness include high information quality and timeliness (Theme 3.1), perceived positive changes after using (Theme 3.2), emotional validation (Theme 3.3), professionalism (Theme 3.4), and expressive freedom (Theme 3.5). Reasons for perceived unhelpfulness include the absence of human presence and the lack of deep emotional engagement (Theme 3.6), inadequate information quality (Theme 3.7), and the lack of professionalism (Theme 3.8).

#### Theme 3.1: High Information Quality and Timeliness

Many users indicated that ChatGPT was effective in EMS because the responses were perceived as informative, comprehensive, accurate, and actionable. In addition, the delivery of the responses was instantaneous, allowing users to obtain information in a timely fashion when they needed it most.

*ChatGPT was quick in generating answers, and the responses were very informative and credible*.

[Female, 29 years old]

#### Theme 3.2: Perceived Positive Changes

Many participants indicated that they experienced ChatGPT as helpful for EMS because they experienced changes in their feelings, behaviors, perspectives, or relationship quality after using ChatGPT or following its advice. For example, they reported connecting better with others, experiencing less emotional distress, changing their perspectives, and taking new actions.

*By turning to ChatGPT, I passed how I was feeling, and it came back with positive results and feedback of the reasons, and put my mind at ease, and I was able to start to control my emotions and thoughts and gain confidence in speaking to my partner and colleagues*

[Female, 50 years old]

*I felt very good after the interaction. ChatGPT was very helpful in my situation. I ended up taking the advice and recommendations, and my average marks improved so much. I am in a better state mentally.*

[Male, 25 years old]

#### Theme 3.3: Emotional Validation and Perceived Care

The emotional and relational quality of ChatGPT’s responses played a key role in users’ perceptions of its effectiveness. Many participants reported that they experienced ChatGPT as highly effective because it was responsive, validating, accepting, and supportive. For some participants, the interaction with ChatGPT felt authentic and human-like; for others, although they did not feel like they were interacting with a real human, they still felt emotionally validated.

*ChatGPT responded not so much like an automated robot but more like a human who genuinely cared about the loss of my father, which actually led me to open up more as I didn’t feel I was being a burden… even though it felt like it a real human wasn’t really behind the messages I was sending it*.

[Male, 20 years old]

*It felt good, even though I wasn’t talking to a human. I felt cared for and listened to, and the responses were very valid and encouraging.*

[Male, 47 years old]

#### Theme 3.4: Professionalism

Some participants reported that they perceived ChatGPT’s responses as professional and reliable for them.

*It helped me a lot, it felt like I was dealing with a professional helper.*

[Female, 29 years old]

#### Theme 3.5: Expressive Freedom

Some users valued the fact that ChatGPT provided them with a responsive channel to freely express without burdening others.

*I think it was very helpful and effective, because it generates a response right away, there’s no feeling of being a burden to another person, and I could simply tell it how I wanted to proceed….*

[Female, 32 years old]

*It was nice to get a response to my questions that I otherwise wouldn't share with anyone else.*

[Male, 25 years old]

#### Theme 3.6: Superficial Emotional Engagement and the Absence of Human Presence

While some participants experienced the interaction as human-like, other participants considered ChatGPT as ineffective for EMS because it did not provide real human contact. They also experienced ChatGPT’s response as lacking a real understanding of emotions, which made the support less effective.

*Answers were helpful but kind of neutral……Even if chat wrote some supportive words and tried to be empathetic, I still had a feeling that it was not a real person and it couldn’t understand how I really feel.*

[Female, 29 years old]

*The first time, ChatGPT produced a very cold and distant response; this response hurt me and was invalidating. Another time, it still responded in a cold, but less unfriendly manner. However, the feeling of superficiality and emotional disconnection in the interaction demonstrated that ChatGPT is not a good tool for seeking a minimum of polite and emotional support.*

[Female, 28 years old]

#### Theme 3.7: Inadequate Information Quality or Limited Personalization

Individuals experienced ChatGPT as ineffective for EMS because of inadequate information quality or limited personalization: they reported that its responses were excessive, repetitive, superfluous, or inappropriate. Additionally, many participants indicated that the responses were too generic and not tailored to their situation.

*I felt unmotivated to change because ChatGPT provided a very general response, not personalized to me and my problem. It wasn’t effective. It informed me that it cannot help me with medical issues. I tried to roleplay with it, with ChatGPT being a therapist, but it didn’t turn out helpful either, as the answers were really general and obvious.*

[Female, 22 years old]

#### Theme 3.8: Lack of Professionalism and Regulatory Rigidity

Some individuals found ChatGPT ineffective due to its lack of professionalism, especially when compared to interacting with mental health professionals. Additionally, they described its responses as rigid and limited. This was especially the case when users encountered the guardrails built into ChatGPT that directed users to seek professional help when they raised sensitive issues like suicidal ideation. It is likely that these users did not perceive the guardrail responses as separate from the AI model. Rather, they likely perceived such responses as the model being cold and official.

*Not helpful at all, only told me to visit a doctor.*

[Male, 24 years old].

*I felt a bit disappointed because ChatGPT gave a cold, “official” response and urged me to seek medical help. It did not help much.*

[Female, 31 years old]

## Discussion

### Principal Findings

This study explored the perspectives of individuals who have been using ChatGPT for EMS through a mixed-methods design. Specifically, we examined the purpose, frequency, and emotional experience of users interacting with ChatGPT for EMS, along with how those users perceived the helpfulness of ChatGPT. We found that ChatGPT is regularly used (typically at least 1 to 2 times per month) by individuals in our sample to meet diverse mental health needs, including traditional mental needs (symptom management, diagnostic evaluation, mental health resources, and literacy) and general psychosocial needs(eg, companionship, relationship guidance, decision facilitation, and well-being enhancement) [14]. Users reported mixed emotional experiences when using ChatGPT for EMS, ranging from positive emotions (feeling emotionally safe, connected, relieved, and grateful), neutral emotions (feeling awkward or skeptically curious), to negative emotions (feeling disappointed, disconnected, or embarrassed). The overall quantitative evaluation was quite positive: almost all users have found ChatGPT at least somewhat helpful. The qualitative data revealed more nuances regarding areas where they found it helpful and unhelpful for EMS. Users attributed its helpfulness to factors that are both crucial for traditional mental health services (eg, perceived changes, emotional connection, and professionalism) and unique for AI modalities (eg, information quality and delivery speed, freedom of expression). The unhelpful aspect was mainly due to its superficial emotional engagement and absence of human presence, inadequate information quality, limited personalization, lack of professionalism, and response rigidity.

Our findings suggest that users seek help from ChatGPT for both reactive and proactive reasons. While a majority of the participants reactively used ChatGPT to obtain relief from distress caused by internal or external stressors, other participants proactively used ChatGPT to improve their general psychosocial needs. Although some of these users may have sought help from ChatGPT because of a lack of access to traditional mental health care, many users purposefully chose to discuss mental health with a nonhuman being, possibly because they trusted it more, thought it provided reliable and objective perspectives, or because they did not want to burden others.

Our study provides insights into the emotional processes of human-GenAI interaction for EMS. Many users described being able to emotionally connect with ChatGPT in a similar way to how they might connect with a human being and felt adequate support and validation. Users also experienced positive emotions such as relief and gratefulness from their use, suggesting GenAI’s potential in facilitating positive emotional changes. Meanwhile, other participants described feeling awkward and skeptically curious, likely reflecting the novelty of seeking help from a nonhuman agent. Importantly, several users reported negative experiences, including disappointment, disconnection, or a sense of emptiness after use, underscoring the limits in support when human contact is absent. The theme of feeling ashamed and embarrassed also emerged, pointing to potential self-judgment or internalized stigma around relying on GenAI rather than human mental health providers. These responses suggest an interesting paradox that future studies should explore: while GenAI may help bypass some barriers linked to stigma around help-seeking behaviors for EMS (eg, fear of human judgment), as also shown in participants’ responses for expressive freedom, it may also introduce a new form of stigma, in which users feel ashamed for secretly seeking support from AI and worried about being judged by the larger society.

Our findings indicate mixed rationales and motivations for use of ChatGPT for EMS among a convenience sample of users who already routinely engage the tool for EMS purposes. These results resonate with previously reported positive experiences of users [8,9], highlighting the key roles of emotional connection and quality advice in shaping users’ perceptions of ChatGPT’s helpfulness for mental health purposes.

These results further highlight an urgent need for researchers, clinicians, and policymakers in the field of mental health to address at least four key questions. First, how effective is GenAI in alleviating psychological distress for individuals with mental health problems and in enhancing the well-being of individuals who use it as a means of proactive coping? Our study suggests that many users have positive perceptions of GenAI for EMS. However, the long-term effects of its use, including both potential benefits and harm, have not been examined; concrete recommendations for practice and policy will need to await the availability of more conclusive empirical (and especially experimental) data on the effectiveness of GenAI.

Second, what are the processes and mechanisms that contribute to the perceived attractiveness and potential effectiveness of GenAI in providing EMS? Will traditional factors that are crucial for psychotherapy, such as the therapist-patient relationship, goal setting, and agency, still play a key role in the use of GenAI for mental health? Our study suggests that these traditional common factors underlying effective therapeutic work still matter, as participants endorsed the emotional connection and support as one of the top reasons for ChatGPT’s perceived effectiveness. In instances where ChatGPT referred the users to seek professional help, possibly due to guardrails in the model, users perceived the model as being cold and unempathetic. User agency and personalization were also mentioned as important factors. As a next step, researchers should examine if there are AI-specific factors, such as the speedy delivery of high-quality information, that may play a critical role in enhancing the perceived effectiveness of this type of mental health self-help. Understanding what factors are behind the perceived attractiveness may also help policymakers find ways to mitigate and prevent harm from GenAI.

Third, what is the quality and nature of the “relationship” that users develop with GenAI when using it for EMS? Our results suggest that users experience some affective ambivalence toward this relationship: some users indicated feeling emotionally safe and connected, while others felt embarrassed and awkward about relying on GenAI for emotional needs. Understanding the nature of future AI-human relationships, especially in the therapeutic context, will help us identify key factors that can enhance mental health self-help while preventing an emotional over-reliance on GenAI that may result in more social isolation and decrease in social skills in the real world.

Finally, how can we better understand the relationship between GenAI-assisted mental health care versus traditional mental health care? Consistent with findings from two other studies [8,9], our research showed that when evaluating the helpfulness of GenAI, users typically compared GenAI with traditional therapy. Future studies should quantitatively examine how the use of GenAI for EMS may be perceived similarly or differently, in complementary or competitive ways, compared to traditional mental health services.

Clinically, our results indicate that health care providers must begin to increase their AI literacy and prepare for conversations with clients regarding the use of AI for mental health purposes. AI literacy is also needed for the general public to discern the benefits and potential ethical risks, including but not limited to questions about its effectiveness, data and privacy concerns, problems related to AI hallucinations and algorithm biases, potential emotional exploitation and overreliance, and the lack of traceability and liability, especially for mental health purposes. It is critical for policymakers to take into account the general attitudes of users toward the use of GenAI for mental health in order to develop regulations that are both informative and adequately protective.

Our study has several strengths. First, we obtained a relatively large sample of active users. This sampling approach allowed us to explore a range of lived experiences of users, enhancing the study’s external validity and identifying patterns that may be broadly applied to diverse communities. Another strength of our study was the mixed-methods design, integrating qualitative and quantitative data to provide a rich and nuanced view of the frequency, purposes, emotional experience, and evaluation of current uses of ChatGPT for mental health and emotional support. Such information provides us with an understanding of both the process and outcomes of GenAI self-help.

Nonetheless, we must consider the strengths of our findings in light of the following noteworthy limitations. First, the sample only included English-speaking users, which may limit the generalizability of our conclusions to non-English-speaking users who use GenAI in other languages. Second, because we prioritized the breadth of perspectives, we did not conduct in-depth interviews with our large sample of participants, which may have revealed more nuanced details and information. Third, our study relied on self-report and cross-sectional data and did not examine the long-term effects by multiple informants, which are the important aspects for examining the efficacy and effectiveness of using GenAI for EMS. Fourth, our findings were based on the experiences of users of one GenAI, ChatGPT; as such, they may not be generalizable to user experiences with other GenAI products. Fifth, our participants were drawn solely from the online platform Prolific and attracted users who were motivated to respond to the survey. The sampling strategy limits the generalizability of our findings to individuals who may not share the same demographic, motivational, or behavioral characteristics as the Prolific participant pool. Sixth, when considering country of residency, our sample does not include respondents from Asian countries and low-income countries and thus has limited generalizability for these populations. Another important limitation is that our study examined perceptions of active users, which has limited generalizability to individuals who have not used ChatGPT for EMS. Finally, our data collection was conducted in April 2024; however, the community policies of OpenAI change rapidly, which may influence the experiences of users and limit the generalizability of our findings to users who used different versions of ChatGPT under different community policies.

### Conclusions

This study provides one of the first large-scale, cross-national examinations of why and how individuals turned to GenAI for emotional and mental health support. At a global level, GenAI is likely changing the future of how individuals seek care for mental health and how mental health services deliver care. As GenAI becomes more integrated into everyday life, the boundaries between formal mental health care and informal self-help, or between treating clinical challenges and meeting daily psychosocial needs, seem to become increasingly blurred. Given the popularity and perceived effectiveness of GenAI among users, it is critical for researchers and mental health professionals to understand the relational and emotional dynamics of how individuals interact with GenAI to better identify mechanisms that may lead to either benefits or harm.

## Supplementary material

10.2196/77951 Multimedia Appendix 1 Quote examples for each subtheme.

## Abbreviations

APA: American Psychological Association
EMS: emotional and mental health support
GenAI: generative artificial intelligence
