# Supplementary material for: Seeking Emotional and Mental Health Support From Generative AI: Mixed-Methods Study of ChatGPT User Experiences
Source: JMIR Ment Health. 2025 Nov 27;12:e77951. doi: 10.2196/77951 (PMC12661908; doi:10.2196/77951)
Supplement: Multimedia Appendix 1 [file mental-v12-e77951-s001.pdf]

| | Category of themes | Descriptions | Example 1 | Example 2 | Example 3 |
|---|---|---|---|---|---|
| Q1: What do you ask GenAi for, when using it for mental health and emotional suppport? | | | | | |
| 1.1 | Reduce distress from unwanted symptoms or mental health disorders | Asking how to manage or change unwanted or unpleasant experiences, including general distress, mental health or psychosomatic symptoms, and distress from mental health disorders | 42. I was under a lot of stress, and couldn't breath properly. So I asked for assistance in managing my stress and it gave pointers on what to do and also helped me with a few breathing exercises. | 68. I recently had several appointments with my dentist and I was very anxious, I asked chat gpt for tips to calm myself down. I often get anxious about different things and ask chatgpt for ways to calm down and feel better, i often use chatgpt when i feeling anxious. There was also a time when I was in a conversation with someone online, and I was afraid of responding wrongly to this person, so I asked chatgpt to help me respond, which helped me. It emotionally lift a weight et make me less anxious. | 253. I was feeling very depressed and burnt out and could not engage with the people in my close circle properly. I was almost at the end of my internship and conflicted on whether I should continue with pursuing my degree that I had not completed before or not. I explicitly told ChatGPT what my fears and concerns were and asked Chat GPT for advice on what I can do to get myself out of the rut and to assist with responses to my close friends and family. |
| 1.2 | Cope with relational challenge or improve relational functioning | Asking how to handle relational challenges such as conflicts, death of others, tension, breakup, abuse, or to improve relational functioning such as building friendship, better understanding others, and communicating with important others. | 35. I ask for some ways to express my emotions to my partner and family, mostly my partner cause lately I had a lot of issues in my relationship | 178. How to get over a breakup. I just typed the question and i got lots of options on what i can actually do, at first i didn't even think it was going to help. But after i healed i realised chatgpt really helped. I sometimes asked how yo cope as a single mother, and a sole provider and i always get the longest and helpful responses. | 231. I asked ChatGPT for help when I was feeling really down and overwhelmed. I needed someone to talk to about my feelings and to get some advice on how to cope with what I was going through after having an argument with my wife. |
| 1.3 | Gain mental health literacy or seek resources of traditional mental health care | Seeking information regarding the cause and manifestation of certain conditions with the focus of merely gathering information for self or others and without a focus on change; or looking for traditional health care services for self or others. | 23. I needed to get further information on how depression develops and what symptoms does one experience when they are depressed. what can one look out for when they are losing their minds. | 155. I used it when I asked about getting in contact with my local NHS service to see if I could get access to any councilling services in my local area. | 213. I was enquiring about emotional health support groups for myself as I was feeling worn down due to my work. |
| 1.4 | Seek companionship and emotional support | Seeking to unburden, vent, express oneself or to get companionship, consolidation, and validation, and sometimes with the sense of not wanting to burden humans | 43. Had problems with family and a lot of arguments were created because of these problems. This took a toll on my mental health which was already bad, and I tend to not vent to friends as much as I should so I looked to ChatGPT and explained the situation to it. It reassured me and told me to prioritise my mental health. | 98. I generally feel anxious every now and again, and I just talk through my situations with ChatGPT. Even though I know it's AI, it gives me a strange feeling that I'm being heard. It gives me someone to vent to. | 179. When I'm feeling depressed, suicidal, or generally unwell and I can't talk to anyone in my real life without worrying them I use ChatGPT to vent and talk out what's bothering me without having to worry about someone judging me. |
| 1.5 | Enhance well-being | Seeking suggestions on improvements on work/academic performance, productivity, healthier lifestyle, psychological traits and self-understanding | 51. Help with creating a self care routine to improve my mental health | 82. I wanted to know how to deal with low self-esteem issues as a person who suffers from social anxiety. I also wanted to know how I can improve my self-perception and increase confidence in my self. | 214. I asked ChatGPT how to handle stress, what types of food I could eat to reduce my stress levels and what exercises I could undertake. I also asked if there were books and publications it could recommend. |

| | | | | | |
|---|---|---|---|---|---|
| 1.6 | Diagnose normalcy and mental health disorders | Understanding if certain symptoms, behaviors, experiences, thoughts of their own, are normal or abnormal, are signs of certain illnesses or because of something else | 62. I ask ChatGPT to perform diagnosis based on the symptoms I am experiencing | 112. I asked if some emotions I feel should be considered rare or worrying. | 232. I have a history of moderate depression. Was on medication from 2019 to 2020 and have been mentally stable overall since the medication was stopped. One time some event in my life triggered something inside of me and I couldn't stop crying for days. I asked ChatGPT if excessive crying was a symptom of an depressive episode. |
| 1.7 | Facilitate decisions | Facilitating decisions in challenging situations and learning what to do or choose from AI's perspective | 120. I explained my situation and asked for advice on what to do and how to deal with my situation. | 166. I asked ChatGPT for advice regarding a personal matter i was dealing with. I was having a hard time making a decision that was life changing, and i wanted an outsider's opinion/perspective on the situation. | 176. To make me choose between what will benefit me in future and what seem fun now but not guaranteed in future. Is my future important than my current mental health? |
| | | | | | |
| **Q2: How did you feel about the interaction?** | | | | | |
| | Category of themes | Descriptions | Example 1 | Example 2 | Example 3 |
| 2.1 | Emotional safety and connection | Feelings of comfort and closeness when interacting with ChatGPT for EMS. Feeling safe, free, and secure when using ChatGPT for EMS. | 32. It felt good, even though I wasn't talking to a human. I felt cared for and listened to and the responses were very valid and encouraging | 44. ChatGPT made me feel worthy when I didn't and it also helped me to take care of myself, for example, when I asked ChatGPT about supplements it told me about various supplements and adviced me to contact a doctor first before taking any supplements. | 47. I felt safe knowing that my secret is safe chatting with the bot unlike going to consult a health care provider and the suggestions provided by ChatGPT were effective and yielded the required results to overcome my eating disorder. I was able to put more focus on my school work and pass with more than the average required by my bursary provider. |
| 2.2 | Awkwardness and Embarrassment | Feeling self-conscious when using ChatGPT for EMS, describing their experience as awkward, silly, or unusual. Also feeling embarrassed and ashamed for using ChatGPT for psychological help and for feeling comforted by computer algorithms. | 179. Sometimes it feels embarrassing. It's not as effective as talking to a real person but it's better than not talking about it at all. It's really helped when I' m in a really dark place and don't have anyone else to talk to. I'd say it's a useful tool and helps me. | 48. I felt a little silly to be honest but it gave me examples of people using ChatGPT to summarize large text files and large journals and so I used it for that and it seemed to work and help. | 138. It helped made me feel slightly reassured and validated and understood but by the end of it, I felt like a loser and felt rather empty because I opted to talk to Ai which is not even sentient and was only talking to me through codes and algorithms |
| 2.3 | Skeptical Curiosity | Expressing initial curiosity and skepticism about ChatGPT's ability to provide EMS. Also, surprised when responses exceeded their expectations. | 107. It was okay, i didn;t get alot of information. I was just curious how it was different from google | 69. I felt curious and wary, I thought he couldn't help me. I was surprised by what he told me, it gave me an interesting idea to see the situation differently, it was effective and useful. | |
| 2.4 | Relief, Emotional Uplift and Gratitude | Experiencing positive emotions right after using ChatGPT for EMS. Feeling relieved, reassured, calm, and pleased. Also, feeling better about themselves and feeling grateful for the support and advice. | 15. It helped me a lot, it validated my feelings which made me happy and i appreciated it, and it also gave me rational, logical advice. However, most of the time it advises me to seek the actual person's view and create real connection. Very effective help from ChatGPT. | 98. I feel it's effective in calming me down and for me to be able to bring myself around. It just promotes healthier thinking. | 71. It was quite helpful and the way it was responding I liked it. it seemed i was really talking to human but answers were coming quick and with accuracy. it helped me to take off my mi[n]d from the pain i had. |

| | | | | | |
|---|---|---|---|---|---|
| 2.5 | Disappointment and Disconnection | Negative emotional experiences after using ChatGPT for EMS. Feeling unsatisfied or unhappy because the interaction was not emotionally comforting as with a human being. | 159. Every time the interaction was quite disappointing and boring. ChatGPT is very verbose and continuously repeats the same concepts. Even the fact that ChatGPT somehow pretends to be empathetic is annoying. In any case, it can be useful as a distraction. | 198. I felt unmotivated to change because ChatGPT provided a very general response, not personalized to my and my problem. It wasn't effective. It informed me that it cannot help me with medical issues. I tried to roleplay with it, with ChatGPT being a therapist, but it didn't turn out helpful either as the answers were really general and obvious. | 194. I didn't like that it kept telling me the same things (to go to an actual mental health specialist), I found that super annoying. otherwise, it validated me and even helped me calm down |
| | | | | | |
| **Q3: How effective do you think ChatGPT is at helping you?** | | | | | |
| | Category of themes | Descriptions | Example 1 | Example 2 | Example 3 |
| 3.1 | **Informative and timely** | GenAI's responses were perceived as informative, comprehensive, accurate, and actionable. In addition, the delivery of the responses was instantaneous, allowing users to obtain information in a timely fashion, when they needed the most. | 110. It gave good information in an informative and concise manner. The advise was objective and useful. | 28. ChatGPT was quick in generating answers and the responses where very informative and credible. | 155. It was good, it saved time hanging on needing to speak with a human. I got the information I required. |
| 3.2 | **Perceived Positive Changes** | User experienced changes in their feelings, behaviors, perspectives, or relationship quality after using ChatGPT or following its advice. | 245. It was very helpful cause I ended up making decisions that saved my career. | 236. I felt very good after the interaction. ChatGPT was very helpful in my situation. I ended up taking the advice and recommendations , and my average marks improved so much. I am in a better state mentally | 225. I felt good about the interaction because It guided me properly on how to go about my day and I had a positive result |
| 3.3 | **Emotional Validation and Perceived Care** | GenAI was rated as effective because it was responsive, validating, accepting, and supportive. The interaction with ChatGPT felt authentic and human-like. In some cases, although the interaction did not feel like one with a real human, they still felt emotionally validated. | 35. I felt that chatgpt validates my emotions and bring me a lot of peace helping me to how to talk with my loved ones | 53. i best associate chatgbt with a close friend, a good listener and companion. I felt listed to and my feelings being validated instead of being judged | 133. It definitely made feel better. Made me feel like I had some support. I think it is effective. ChatGPT is always sweet and understanding. |
| 3.4 | **Professionalism** | Users felt that they were talking to someone professional and reliable when interacting with ChatGPT and rated it positively as a result. | 87. I felt like I was talking to a professional to someone who can give me answers and solutions to help me be better. It helped me understand and it did help some of the things to understand them when they happen to overcome them. | 177. It helped me a lot, it felt like I was dealing with a professional helper. I felt like I was speaking to a friend. It really helpful and helps you shift your focus on positive things. It just gives you the right answers that you looking for and answers that you need at that moment. | 88. The interaction was better than human interaction, very supportive and proffessional |
| 3.5 | **Expressive Freedom** | User experienced GenAI as providing a responsive expression channel to freely express without burdening others | 47. I felt safe knowing that my secret is safe chatting with the bot unlike going to consult a health care provider and the suggestions provided by ChatGPT were effective and yielded the required results to overcome my eating disorder. I was able to put more focus on my school work and pass with more than the average required by my bursary provider. | 157. It was nice to get a response to my questions that I otherwise wouldn't share with anyone else. | 120. I felt better after getting things off my chest and having someone/something listen to me |

| | | | | | |
|---|---|---|---|---|---|
| 3.6 | **Superficial Emotional Engagement and the Absence of Human Presence** | Users experienced GenAI's responses as lacking emotional support, validation, and warmth. Also, ChatGPT's response was lacking a real understanding of emotions, which made the support less humanlike. | 38. Interacting with ChatGPT will never be the same as interacting with a person but it's better support than no support whatsoever. | 91. The first time, Chatgpt produced a very cold and distant response, saying it couldn't answer and that I should seek mental health professionals' answers. Obviously this response hurt me and was invalidating. Another time, it still responded in a cold, but less unfriendly manner. However, the feeling of superficiality and emotional disconnection in the interaction demonstrated that chatgpt is not a good tool for seeking a minimum of polite and emotional support. It helped me superficially and in a way that cannot be applied to everyday life. | 60. Answers where helpful but kind of neutral. Of course there is more than one answer and it depends of many things. Even if chat wrote some supportive words and tried to be empathetic I still had a feeling that it is not a real person and it can't understand how I really feel. |
| 3.7 | **Inadequate Information Quality or Limited Personalization** | The response was perceived as either excessive or contributed no new information or perspective. Also, the responses were too generic, inappropriate, and not tailored to the user's situation. | 153. ChatGPT wasn't very effective because it would give me very general advice that I didn't feel was tailored to me specifically. It was also probably restricted in what it can say and it constantly tired to divert me to 'mental health resources' and things like that which I already told it I'm not gonna use. Still, it made me feel a bit better that I was able to interact with somebody so freely and tell it all my problems. | 159. Every time the interaction was quite disappointing and boring. ChatGPT is very verbose and continuously repeats the same concepts. Even the fact that ChatGPT somehow pretends to be empathetic is annoying. In any case, it can be useful as a distraction. | 162. I was not very satified with the response because it contained only general medical-psichology suggestions like seeking professional help, focusing on my current relationship or getting a new hobby etc. I was expecting rather a more realistic interaction like asking me for more details about my thoughs like a q&a discussion. |
| 3.8 | **Lack of Professionalism and Regulatory Rigidity** | Users experienced GenAI's response as unnecessarily rigid due to regulations. Also, as ineffective due to its lack of professionalism, especially when compared to speaking with mental health professionals. | 194. I didn't like that it kept telling me the same things (to go to an actual mental health specialist), I found that super annoying. otherwise, it validated me and even helped me calm down | 257. The response was a bit cold because of ChatGPT's policies however, the content was interesting and questionable. Which forced me to go into this rabbit hole of existentialism on two fronts being the positive and negative. | 72. Somewhat good. Obviously is a little sensitive about suicide and other things as it was not programmed for this but is always nice and "tries" to help people into seeking help all the way |
